# Advances in Natural Fibers and Polymers

**DOI:** 10.3390/ma14102607

**Published:** 2021-05-17

**Authors:** Francesc X. Espinach

**Affiliations:** LEPAMAP+PRODIS Research Group, Development and Product Innovation, University of Girona, Maria Aurèlia Capmany, 61, 17003 Girona, Spain; Francisco.espinach@udg.edu

The use of natural fibers as reinforcement for polymer-based composites has been attracting the interest of the scientific community for a long time. Despite the maturity of natural fiber-reinforced polymers, such materials continue to arouse the interest of researchers.

On the one hand, the environmental concern of society has increased consistently during recent decades. This has caused growing attention to the impact of consumerism on the environment and the birth of laws and regulations promoted by national governments and international organizations. A clear example is the 2030 Agenda for Sustainable Development, promoted by the United Nations and signed by more than 190 countries. In this agenda, there are specific goals devoted to plastic recycling (goal 12) and to avoiding the use of plastic bags to keep the oceans clean (goal 14).

On the other hand, the use of natural fibers instead of mineral fibers has some advantages. Natural fibers are less abrasive and thus avoid the deterioration of equipment. Natural fibers are less dense than mineral fibers and their composites are lighter, providing better specific properties. This is of interest for automotive and aerospace industries, always seeking to reduce the weight of their vehicles. Additionally, natural fibers are safe to manipulate and, unlike mineral reinforcements, like glass fibers, are not harmful to human beings.

Lignocellulosic fibers can be obtained from wood, annual plants, as waste from agroforestry, or as a byproduct of some industrial processes, like textiles or papermaking. The possibility of using agroforestry waste as reinforcement for polymer-based composites can increase its value chain, creating richness by adding value to waste that is usually incinerated in the field. The use of byproducts can prevent disposal in landfills and the associated contamination. Other sources for natural fibers are biological, like air or feathers, which can be mixed with a matrix to obtain composite materials.

Nonetheless, there are some questions worth researching, to normalize the use of natural fibers instead of mineral ones. The use of hydrophilic natural fibers with hydrophobic matrices implies obtaining weak interphases that harm the potential mechanical properties of the composites. This is a very active field of research, which has achieved multiple solutions but must continue to be active, to obtain interphases that ensure a good combination between the tensile and impact properties of the materials. Moreover, natural fibers show a wider variation in their intrinsic properties than human-made reinforcements, decreasing the certainty of the composite material properties, which can vary from one batch to the other.

Another interesting field of research is the use of bio-based polymer as a matrix for natural fiber-reinforced composites. These matrices are oil independent and come from renewable resources. To date, its main drawback is that its cost is higher than that of oil-derived plastics, but bio-based polymers like poly(lactic acid) have a noticeably increased presence in the market. Bio-based plastics must show competitive properties in comparison to oil-derived polymers to be of interest to the industry. The use of natural fibers can support their use, by increasing their properties, decreasing the cost, and decreasing the environmental impact of the materials.

This Special Issue includes studies devoted to the study of the properties, uses, and impacts of natural fiber-reinforced composites.

The scientific topics addressed in this issue are summarized as follows:

- Annual plants
- Recycled strands
- Natural fiber composites
- Life cycle assessment
- Mechanical properties
- Interphase
- Micromechanics
- Biodegradable matrices
- Bio-based polymers

In brief, the scientific papers published in this thematic issue on advances in natural fibers and polymers are devoted to the following aspects.

In their study “Conductive Regenerated Cellulose Fibers for Multi-Functional Composites: Mechanical and Structural Investigation” [1], Zainab Al-Maqdasi et al. investigated the mechanical properties of regenerated cellulose fibers coated with copper via an electroless plating process. They also researched the molecular structure changes and suitability for use in sensing applications of the materials. The results indicated that regenerated cellulose fibers coated with copper show potential when combined with conventional composites of glass or carbon fibers as structure monitoring devices without significantly affecting their mechanical performance.

Xiaoxiao Zhang et al. devoted their paper “Reinforcing Mechanisms of Coir Fibers in Light-Weight Aggregate Concrete” [2] to the use of natural fibers from agricultural wastes, such as coir fibers as an alternative reinforcement in concrete composites. The goal of the research was to investigate the effects of coir fibers on the hydration reaction, microstructure, shrinkages, and mechanical properties of cement-based lightweight aggregate concrete (LWAC), concluding that treatments with coir fibers promote the hydration reaction of cement due to the accelerating effect of the different treating agents.

Riko Šafarič et al. published a paper titled “Preparation and Characterisation of Waste Poultry Feathers Composite Fibreboards” [3]. The growth of poultry meat production increases feather waste quantities every year. The researchers produced composite fiberboards with different percentages of wood and feathers, testing their mechanical properties. The obtained materials showed notable soundproofing properties and cost advantages.

Philipp Sauerbier et al. published a paper titled “Surface Activation of Polylactic Acid-Based Wood-Plastic Composite by Atmospheric Pressure Plasma Treatment” [4]. They produced composites based on polylactic acid (PLA) and investigated the influence of a dielectric barrier discharge (DBD) plasma treatment on the composites. The research revealed that after the treatment, there was a surface roughening, and contact angles decreased noticeably.

Ling Pan et al. wrote a paper titled “Preparation and Properties of Microcrystalline Cellulose/Fish Gelatin Composite Film” [5]. They used fish gelatin to prepare microcrystalline cellulose-based films. The researchers used a cross-linking agent to increase the interactions between microcrystalline cellulose and fish gelatin. The obtained results showed that the developed films were suitable for use in the biomedical field.

Dragan Kusić et al., in their research, titled “Thermal and Mechanical Characterization of Banana Fiber Reinforced Composites for Its Application in Injection Molding” [6], characterized composite materials based on high-impact polystyrene (HIPS), acrylonitrile butadiene styrene (ABS), and high-density polyethylene (HDPE) matrices, reinforced with short fibers of plantain. The results showed increases in the tensile and flexural properties when the natural fibers were added to the composites and the suitability of such waste fibers as polymer reinforcement.

Jerzy Korol et al. published “Comparative Analysis of Carbon, Ecological, and Water Footprints of Polypropylene-Based Composites Filled with Cotton, Jute and Kenaf Fibers” [7]. They studied the carbon, ecological, and water footprint assessment of polypropylene-based composites filled with cotton, jute, and kenaf fibers. The paper helped in understanding all the issues affecting the environmental impact of composite materials and avoiding unsubstantiated or preconceived conclusions.

Stefan Cichosz and Anna Masek contributed two papers to the Special Issue: “Superiority of Cellulose Non-Solvent Chemical Modification over Solvent-Involving Treatment: Solution for Green Chemistry (Part I)” [8], and “Superiority of Cellulose Non-Solvent Chemical Modification over Solvent-Involving Treatment: Application in Polymer Composite (part II)” [9]. In Part I, the researchers modified cellulose with a process that did not incorporate any solvent. The presented process fulfills green chemistry requirements and avoids using toxic solvents. Part II used cellulose fibers obtained by solvent and non-solvent processes as reinforcement. The analysis of the results showed the advantages of reinforcements obtained without solvents, in terms of mechanical properties and environmental impact.

Marc Delgado-Aguilar et al. published “Polylactic Acid/Polycaprolactone Blends: On the Path to Circular Economy, Substituting Single-Use Commodity Plastic Products” [10]. The authors studied the possibility of substituting oil-based polymers with bio-based plastics. The authors studied the micromechanics of the interphase between the matrix and the reinforcements and the influence of the percentage of fibers on the mechanical properties of the composites. The paper shows the suitability of bio-based plastic composites compared to oil-based composite commodities.

David Hernández Díaz et al. published “Impact Properties and Water Uptake Behavior of Old Newspaper Recycled Fibers-Reinforced Polypropylene Composites” [11]. The authors used fibers from recycled newspapers and researched the impact and water uptake properties of the composites. The composites showed impact strength and water uptake behaviors that compared positively with materials reinforced with raw lignocellulosic fibers.

In another paper, David Hernández Díaz et al. presented a graphic approach to the mechanical properties of composite materials. The article “Topography of the Interfacial Shear Strength and the Mean Intrinsic Tensile Strength of Hemp Fibers as a Reinforcement of Polypropylene” [12] features topography terrain display techniques to study the evolution of the mechanical properties of the composites against two factors. This graphical approach identified areas sensitive to the variation of one of the factors, especially the presence of coupling agents.

Elsadig Mahdi and Aamir Dean published “The Effect of Filler Content on the Tensile Behavior of Polypropylene/Cotton Fiber and poly(vinyl chloride)/Cotton Fiber Composites” [13]. The paper shows the impact of filler content on the mechanical properties of cotton fiber on PP- and PVC-based composites under quasi-static loading. The results showed the impact of the filler on the ability of the composites to absorb energy and the failure mechanism of the materials.

Mario D. Monzon et al. published “Experimental Analysis and Simulation of Novel Technical Textile Reinforced Composite of Banana Fibre” [14]. The authors characterized a technical textile made from enzyme-treated banana fibers. The paper presents a methodology to preview the behavior of the textiles when applied to real parts, with a 9% maximum error.

Maria Cristina Righetti et al. presented the paper “Thermal, Mechanical, Viscoelastic and Morphological Properties of Poly(lactic acid) based Biocomposites with Potato Pulp Powder Treated with Waxes” [15]. The treatment of potato pulp particles with oil-based waxes improved the strength of the interphase with the PLA matrix. This methodology expands the possible use of biocomposites, reducing the cost of the products and favoring a circular economy.

The contributions of the authors to this Special Issue on advances in natural fibers and polymers can be valuable for researchers, engineers, architects, designers, and other practitioners involved in the design and use of environmentally friendly materials. The papers show the interest in the natural fiber-reinforced polymer field of research and the opportunities for further research. There is much to research to show the environmental impact of natural fiber-reinforced composites compared commodity materials, and the development of materials able to be used for structural purposes.

I would like to express my gratitude to all the authors that have contributed to the Special Issue and all the reviewers involved in the process for their generous work.

I must thank the *Materials* Editorial Board for allowing me to edit this Special Issue, and for their support and patience.
